# Evaluation of a Deep Learning Algorithm for Automated Spleen Segmentation in Patients with Conditions Directly or Indirectly Affecting the Spleen

**DOI:** 10.3390/tomography7040078

**Published:** 2021-12-13

**Authors:** Aymen Meddeb, Tabea Kossen, Keno K. Bressem, Bernd Hamm, Sebastian N. Nagel

**Affiliations:** 1 Charité—Universitätsmedizin Berlin, Corporate Member of Freie Universität Berlin and Humboldt-Universität zu Berlin, Klinik für Radiologie, Hindenburgdamm 30, 12203 Berlin, Germany; keno-kyrill.bressem@charite.de (K.K.B.); bernd.hamm@charite.de (B.H.); sebastian.nagel@charite.de (S.N.N.); 2 CLAIM—Charité Lab for AI in Medicine, Charité—Universitätsmedizin Berlin, Augustenburger Platz 1, 13353 Berlin, Germany; tabea.kossen@charite.de; 3 Berlin Institute of Health, Charité—Universitätsmedizin Berlin, Charitéplatz 1, 10117 Berlin, Germany

**Keywords:** automated segmentation, deep learning, image processing, diagnostic techniques and procedures, diagnosis

## Abstract

The aim of this study was to develop a deep learning-based algorithm for fully automated spleen segmentation using CT images and to evaluate the performance in conditions directly or indirectly affecting the spleen (e.g., splenomegaly, ascites). For this, a 3D U-Net was trained on an in-house dataset (n = 61) including diseases with and without splenic involvement (in-house U-Net), and an open-source dataset from the Medical Segmentation Decathlon (open dataset, n = 61) without splenic abnormalities (open U-Net). Both datasets were split into a training (n = 32.52%), a validation (n = 9.15%) and a testing dataset (n = 20.33%). The segmentation performances of the two models were measured using four established metrics, including the Dice Similarity Coefficient (DSC). On the open test dataset, the in-house and open U-Net achieved a mean DSC of 0.906 and 0.897 respectively (*p* = 0.526). On the in-house test dataset, the in-house U-Net achieved a mean DSC of 0.941, whereas the open U-Net obtained a mean DSC of 0.648 (*p* < 0.001), showing very poor segmentation results in patients with abnormalities in or surrounding the spleen. Thus, for reliable, fully automated spleen segmentation in clinical routine, the training dataset of a deep learning-based algorithm should include conditions that directly or indirectly affect the spleen.

## 1. Introduction

The spleen is the largest lymphoid organ and plays a significant role in the immune response [1]. It can be affected by hematological malignancies, infections and other systemic diseases, leading to changes in volume, morphology, and metabolic activity [2,3,4,5,6]. Computed Tomography (CT) has been shown to be the most reliable noninvasive method for the volume measurement and assessment of splenic involvement in various diseases [7,8]. A precise segmentation of the spleen can deliver valuable information about morphological changes, but manual segmentation of the spleen is time-consuming and not feasible in clinical routine. Several automatic and semi-automatic methods based on image processing have been proposed for abdominal organ segmentation [7,9,10]. However, the accuracy of these methods is often limited, and manual correction is required [11].

In recent years, deep learning algorithms have achieved a high performance in semantic segmentation tasks [12,13,14,15]. A widely used deep neural network to segment structures and organs in medical images is the U-Net [16]. The U-Net represents a symmetric Convolutional Neural Network (CNN) consisting of two parts, a down-sampling path of convolutions which extracts image information, followed by an up-sampling path of convolutions to produce pixel- or voxel-wise predictions [17]. Skip-connections from down-sampling to up-sampling blocks are used within the U-Net architecture to preserve spatial information [17].

Earlier studies reported accurate results of spleen segmentation using CNN algorithms on CT-images [18,19]. However, these studies focused on technical feasibility without validating the algorithms in varying conditions affecting the spleen [20,21].

The aim of this study was to develop a deep learning-based automatic segmentation model that correctly segments the spleen, even under diverse conditions. For this, a dataset consisting of patients with different medical conditions, with or without splenic involvement, was curated. To further assess the effects of the training dataset on the performance, the model was also trained using another dataset of patients with an unremarkable spleen.

## 2. Materials and Methods

This retrospective study was approved by our institutional review board (No.: EA4/136/21). The requirement for informed consent was waived due to the retrospective design of the study.

The deep learning-based automatic segmentation model was developed on the open-source MONAI Framework (Medical Open Network for AI, version 0.5.0) [22]. During the first stage, one model was trained on an in-house dataset consisting of patients with different conditions with or without splenic involvement (e.g., splenomegaly, ascites). During the second stage, another model was trained on an open medical image dataset comprising patients with an unremarkable spleen (Medical Segmentation Decathlon) [23]. Both models were of the same architecture. The performance of the two segmentation models was then evaluated to assess whether algorithms trained on unremarkable organs could also be applied in patients with alterations that change or obscure the normal splenic anatomy, and vice versa.

### 2.1. In-House Dataset

We retrospectively identified 61 consecutive CT scans covering the abdomen in portal venous phase of patients with different underlying conditions, either with or without splenic involvement (search period from October 2020 to March 2021). The number of CT scans was set at 61 to match the number of patients in the open dataset as described below. CT scanners from two manufacturers were used to acquire the CT scans: Aquilion One (number of performed examinations = 24) and Aquilion PRIME (n = 18) from Canon Medical Systems (Otawara, Tochigi, Japan) and Revolution HD (n = 5), Revolution EVO (n = 8) and LightSpeed VCT (n = 6) from General Electric Healthcare (Boston, MA, USA). The contrast agents used were iomeprol (Imeron^®^, Bracco Imaging, Milan, Italy) iobitridol (Xenetix, Guerbert, Villepinte, France) and iopromide (Ultravist, Bayer, Leverkusen, Germany) with amounts varying between 100 and 140 mL. Portal venous phase imaging was performed at 70–80 seconds after intravenous administration of the contrast agent. Axial reconstructions with a slice thickness of 5 mm without gaps were used in this study.

The dataset was subsequently curated by two radiologists (R1, with 9 years and R2 with 5 years of experience) to ascertain sufficient image quality (CT scans with visually sharp depiction of the splenic contour and optimal portal venous phase, no motion artifacts). After curation, the CT studies were extracted from the PACS (Picture Achieving and Communication System) and de-identified by anonymization of the Digital Imaging and Communication in Medicine (DICOM) tags. The spleen was subsequently semi-automatically segmented using a 3D Slicer (Version 4.11.20210226, http://www.slicer.org (accessed on 29 October 2021)) [24]. Contours were manually adjusted by the two radiologists. The Intraclass Correlation Coefficient (ICC) Estimate between the two radiologists was greater than 0.9 (95% CI: 0.914–0.998) and was indicative of excellent reliability [25]. The segmentations of R2 were considered as the ground truth (GT) for the training and testing of the U-Net model (as described below). Patient and disease characteristics are outlined in Table 1.

### 2.2. Medical Segmentation Decathlon Dataset (Open Dataset) 

The Medical Segmentation Decathlon (MSD) is a web-based open challenge to test the generalizability of machine learning algorithms applied to segmentation tasks [23]. Spleen segmentation is one task of the MSD for which a dataset is provided. The spleen dataset contains 61 CT studies—41 studies for training and validation, 20 studies for testing—of patients undergoing chemotherapy treatment for liver metastases at Memorial Sloan Kettering Cancer Center (New York, NY, USA). Since the challenge is still ongoing, the ground truth of the test dataset was not publicly available and therefore the segmentation was performed by our radiologists as described above using a 3D Slicer.

### 2.3. Image Preprocessing and Model Architecture 

The image data were reformatted from standard DICOM to Neuroimaging Informatics Technology Initiative (NIfTI) format and subsequently transferred to an in-house server for training, validation, and testing of the model.

The 3D U-Net Model was implemented using the Python programming language (version 3.7, Python Software Foundation, https://www.python.org (accessed on 12 April 2021)) on the open-source deep learning framework MONAI in conjunction with the PyTorch Lightning framework (version 0.9.0, https://www.pytorchlightning.ai (accessed on 12 April 2021)) and PyTorch (version 1.8.1 https://pytorch.org (accessed on 12 April 2021)) [26], Numpy (version 1.19.5 https://numpy.org (accessed on 12 April 2021)) [27] as well as Matplotlib (version 3.0.0 https://matplotlib.org (accessed on 12 April 2021)) [28] libraries.

The model architecture consisted of an enhanced version of U-Net which has residual units, as described by Kerfoot et al. [23]. Each layer has an encode and decode path with a skip connection between them. In the encode path, data were down-sampled using strided convolutions and in the decode path they were up-sampled using strided transpose convolutions. During training, we used the Dice loss as the loss function and Adam as the optimizer, with a learning rate set at 1e-4 and backpropagation to compute the gradient of the loss function. 

### 2.4. Network Training

**Figure 1:**
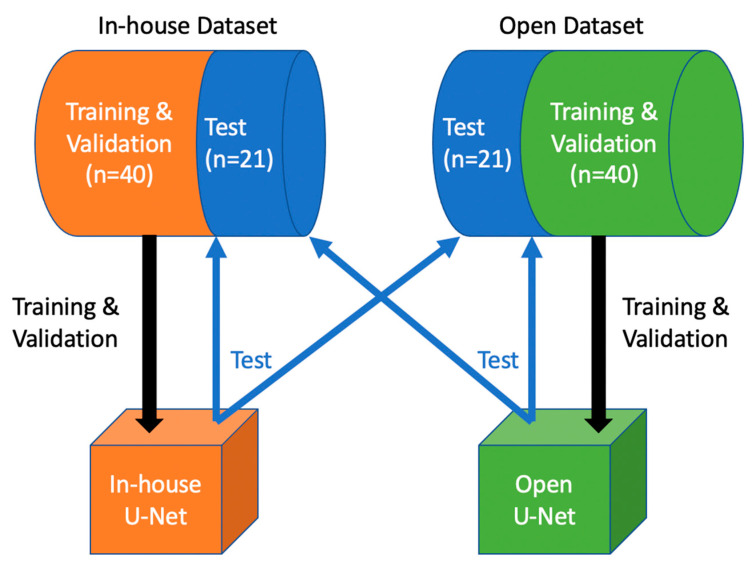
Study Design: In-house U-Net was trained and validated with the in-house training and validation dataset, then tested on both test sets. Open U-Net was trained and validated with the open training and validation dataset, then tested on both test sets.

The in-house and open dataset (each n = 61) were both split into a training (n = 32.52%), a validation (n = 9.15%) and a test dataset (n = 20.33%). First, we set deterministic training for reproducibility. In-house U-Net was trained and validated with the in-house dataset, and subsequently tested on the open test dataset. Open U-Net was trained and validated with the open dataset, and tested on the in-house test dataset. Figure 1 depicts the design of our study. For training we used a batch size of 2. Each model was trained for 500 epochs and validated every two epochs. During training, we used different augmentations: image data were resampled to a voxel size of 1.5 × 1.5 × 2.0 mm (x, y and z direction) using bilinear (images) and nearest neighbor interpolation (segmentation masks). The images were then windowed and values outside the intensity range of −57 to 164 Hounsfield units were clipped to discard unnecessary data for this task. Because of the large memory footprint of the 3D training model, each scan was randomly cropped to a batch of balanced image patch samples based on a positive/negative ratio with a patch size of 96 × 96 × 96 voxels.

### 2.5. Image Postprocessing

To produce the final segmentation results, various post-processing transforms were applied: A sigmoid activation layer was added. Since each patch was processed separately, the results were stitched together, and converted to discrete values with a threshold set at 0.5 to obtain binary results. The output was then resampled back to the original scan resolution. Subsequently, the connected components were analyzed and only the largest connected component was retained. For a better visualization of the results, the contour of segmentation was extracted using Laplace Edge detection and was merged with the original image.

### 2.6. Statistical Analysis and Evaluation

Both models were evaluated on the in-house and the open test dataset. Four established segmentation metrics were used: Dice similarity coefficient, Hausdorff distance, average symmetric surface distance and relative absolute volume difference. The Dice similarity coefficient (DSC) provides information about the overlapping parts of segmented and ground truth volumes (1 for a perfect segmentation, 0 for the worst case), and is defined as 2 × true positive voxels/(2 × true positive voxels + false negative voxels). The maximum Hausdorff distance calculates the maximum distance between two point sets (in our case voxels, 0 mm for a perfect segmentation, maximal distance of image for the worst case). The average symmetric surface distance (ASSD) determines the average difference between the surface of the segmented object and the reference in 3D (0 mm for a perfect segmentation, maximal distance of image for the worst case). The relative absolute volume difference (RAVD) provides information about the differences between volumes of segmentation and the ground truth (0% for a perfect segmentation, 100% for the worst case). Per metric, the mean, standard deviation and 95% confidence intervals were reported. We computed the *p*-values using the Mann–Whitney U test to evaluate whether there was a statistical difference between the DSC of the in-house U-Net and the open U-Net. A *p*-value below 0.05 was considered to indicate statistical significance. Statistical analysis was performed using Python 3.7, the scikit-learn library (version 0.23.1, https://scikit-learn.org (accessed on 6 June 2021)) [29] and statsmodels (version 0.11.1, https://www.statsmodels.org (accessed on 6 June 2021)) [30].

## 3. Results

### 3.1. Segmentation Performance in the Open Test Dataset

On the open dataset, the in-house U-Net obtained a DSC of 0.906 ± 0.071 and the open U-Net a DSC of 0.897 ± 0.082. The surface distance-based metrics (maximum Hausdorff and ASSD) showed that open U-Net had fewer outliers than the in-house U-Net and thus had a better segmentation result. The relative absolute volume difference (RAVD) for in-house U-Net and open U-Net were 9.70% and 11.49% respectively. The Mann–Whitney U test showed no significant difference between the DSC of the in-house U-Net and the open U-Net (*p* = 0.526), as described in Table 2.

### 3.2. Segmentation Performance in the In-House Test Dataset

In the in-house dataset, the in-house U-Net obtained a DSC of 0.941 ± 0.021 and the open U-Net a DSC of 0.648 ± 0.289. The surface distance-based metrics (maximum Hausdorff and ASSD) showed that open U-Net had many more outliers than the in-house U-Net. The relative absolute volume difference (RAVD) for in-house U-Net and open U-Net were 4.20% and 42.25% respectively. The Mann–Whitney U test showed a significant difference between the DSC of the in-house U-Net and the open U-Net (*p* < 0.001).

**Figure 2:**
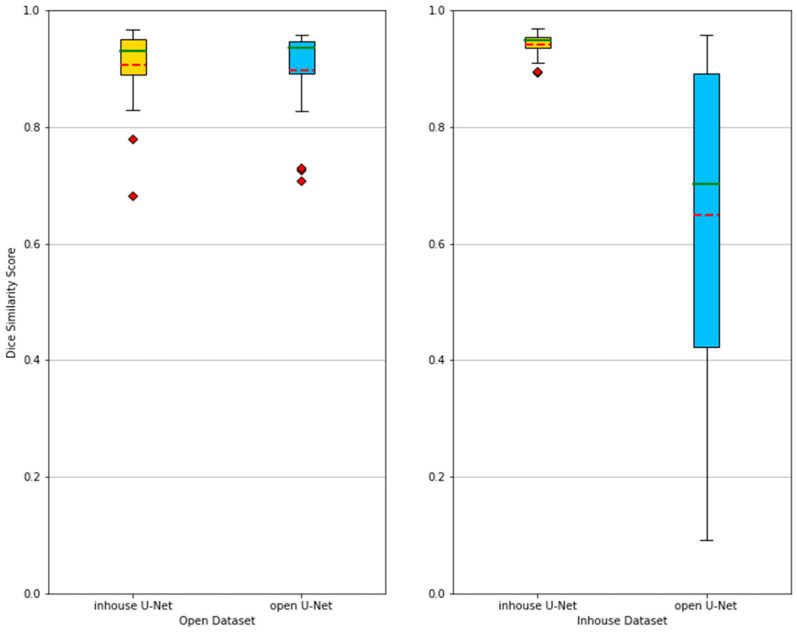
Boxplots showing the segmentation performances of in-house U-Net and open U-Net applied on the open and in-house test datasets. The methods are compared using the Dice similarity score (DSC). Mean (red dashed) and median (green) values are depicted. When applied to the in-house test dataset, the performance of the open U-Net generally drops and becomes more unreliable, which is depicted by a lower mean and median, as well as a larger spread between the best and worst DSC. Table 2 further gives an overview of the results.

**Figure 3:**
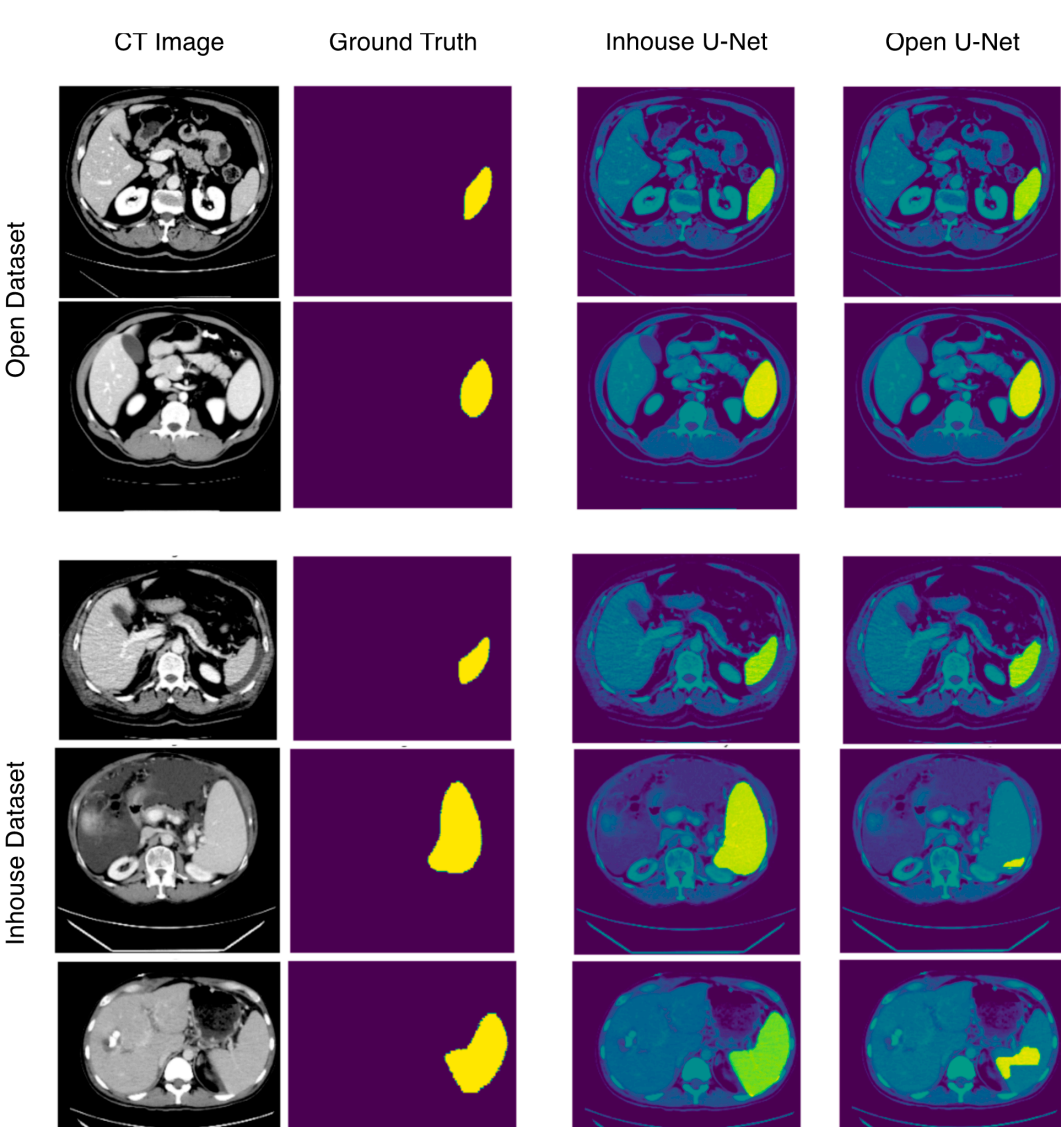
Sample images showing segmentation results of in-house and open U-Net using the in-house and open datasets. In the open dataset, in-house U-Net and open U-Net show performances, whereas in the in-house dataset, open U-Net shows bad segmentation results, especially in patients with splenomegaly.

On the basis of these results (Table 2), the in-house U-Net outperformed the open U-Net in all four metrics in the in-house test set, and the difference was highest in patients presenting abnormalities within or surrounding the spleen. The in-house U-Net furthermore outperformed the open U-Net also in the open test set on two metrics (DSC and RAVD) but showed worse results in the surface distance-based metrics (ASSD and maximum Hausdorff). There was no significant statistical difference of the segmentation results based on the DSC. The Figure 2 shows boxplots comparing the segmentation performance of the models on both testing datasets. Figure 3 shows samples of a qualitative evaluation of segmentation results of the two models.

## 4. Discussion

In this study, we evaluated an open-source deep learning-based algorithm to automatically segment the spleen in CT scans of patients with or without splenic abnormalities. When trained and tested on patients with an unremarkable spleen (open dataset), the U-Net showed accurate segmentation results. However, the segmentation accuracy decreased when the model was evaluated on a dataset including CT scans with alterations in the splenic anatomy or abnormalities in the neighboring structures. 

A variety of methods have been proposed for an automated segmentation of abdominal organs, including statistical shape models [31], atlas-based models [10], and three-dimensional deformable models [32]. These methods showed acceptable segmentation performances, but often needed manual correction [11]. Now, deep learning-based segmentation methods are rapidly overtaking classical approaches for abdominal organ segmentation [33]. For example, the methods of Gibson et al., Yura Ahn et al. and Gabriel et al. reached a mean DSC over 0.95 in automated segmentation of the spleen [19,34,35]. However, Gabriel et al. reported bad and failed segmentation results in patients with splenic distortions, even if their model reached a DSC of 0.962 [34]. Table 3 shows that not all methods were assessed considering splenic abnormalities.

The aim of this study was to develop a robust deep learning algorithm for spleen segmentation across various conditions that can alter or obscure the normal splenic anatomy. The segmentation results showed clearly that a robust segmentation algorithm needs to be trained with a dataset including different conditions affecting the spleen in order to produce reliable results appropriate for clinical routine.

There are multiple potential applications of such an algorithm in clinical practice. For example, automated precise segmentation and feature extraction could help to identify quantitative imaging biomarkers to differentiate between toxic, infectious and hematological causes of splenomegaly, or to assess the severity of cirrhotic liver diseases and portal hypertension, as suggested by previous studies [39,40]. The potential clinical implications of our algorithm should thus be evaluated in future studies in this regard.

Although our study showed reliable results, it had several limitations. First, our in-house dataset was small. This was intended to match the medical segmentation decathlon dataset (n = 61) and thus to receive comparable results. Yura Ahn et al. and Gabriel et al. trained their algorithms with 250 and 450 manually segmented CT-Scans, respectively [34,35]. In addition, no cross-validation was performed for training. This could have improved the segmentation performance on the validation dataset, but it often leads to model overfitting. Furthermore, our 3D U-Net was trained and validated using only portal venous phase CT images. To achieve accurate results on different acquisition techniques, our algorithm may need additional training data and updated model weights using Transfer Learning [41]. Moreover, complex intrasplenic distortions, such as splenic infarctions or focal lesions, were excluded from this study due to their relatively low incidence and high heterogeneity, creating the risk of a preselection bias. To overcome the scarcity of such distortions, data augmentation using Generative Adversarial Networks (GANs) could be investigated in future research [42]. 

## 5. Conclusions

We trained and evaluated the performance of state-of-the-art deep learning-based algorithms for an automated spleen segmentation in patients with diseases with and without splenic involvement and could demonstrate the crucial role of the quality of the training dataset. In order to achieve highly accurate segmentation results in clinical routine, the training dataset should include an important proportion of patients with splenic abnormalities. Future studies are needed to investigate the role of data augmentation using GANs to compensate the low incidence of rare conditions and the role of transfer learning to avoid the intensive time and energy-consuming training of the algorithm.

## Figures and Tables

**Table 1:** Characteristics of the In-house Dataset.

	Training and Validation Dataset	Testing Dataset
Number of Patients	41	20
Female	23 (56%)	5 (25%)
Age *	62.6 ± 16.2	60.4 ± 15.1
Splenomegaly	21 (51.2%)	7 (35%)
Liver cirrhosis	11 (26.8%)	3 (15%)
Lymphoma	6 (15.6%)	2 (10%)
No pathology	4 (9.8%)	1 (5%)
Other **	21 (51.2%)	14 (70%)

Unless otherwise indicated, data are expressed as number of participants. * Data are expressed as mean ± standard deviation; ** other pathologies include lung, pancreas, liver, prostate, and colorectal cancers, without direct splenic involvement, but in whom splenomegaly or ascites could be present.

**Table 2:** Model performances on the in-house and open testing datasets.

Model	Dataset		DSC	RAVD * (%)	ASSD (mm)	Hausdorff (mm)
In-house U-Net	In-house testing dataset	Mean ±SD	0.941 ±0.021	4.203	0.772 ±0.274	7.137 ±5.440
		95% CI	0.932–0.951	2.313–6.094	0.644–0.900	4.591–9.683
	Open testing dataset	Mean ±SD	0.906 ±0.071	9.690	0.999 ±0.657	8.787 ±6.889
		95% CI	0.873–0.939	3.877–15.504	0.692–1.307	5.563–12.011
Open U-Net	In-house testing dataset	Mean ±SD	0.648 ±0.289	42.255	5.158 ±5.881	30.085 ±30.885
		95% CI	0.513–0.784	26.503–58.008	2.406–7.911	15.630–44.539
	Open testing dataset	Mean ±SD	0.897 ±0.082	11.488	0.982 ±0.618	7.569 ±5.242
		95% CI	0.859–0.935	5.323–17.653	0.693–1.272	5.115–10.023

SD: standard deviation. 95% CI: 95% confidence interval. DSC: Dice Similarity Score (the higher the better). RAVD: Relative Absolute Volume Difference (the lower the better); * no standard deviation is reported because this metric is zero centered and we used the absolute value. ASSD: Average Symmetric Surface Distance (the lower the better). Hausdorff: Maximum Hausdorff Distance (the lower the better).

**Table 3:** Comparison between our in-house U-Net (**in Bold**) and previous works.

Method	DSC	Modality	Abnormalities
Gauriau et al. [36]	0.870 ± 0.150	Abd. CT	No
Wood et al. [37]	0.873	Abd. CT	Yes
Gloger et al. [38]	0.906 ± 0.037	MRI	No
**In-house U-Net**	**0.941 ± 0.021**	**Abd. CT**	**Yes**
Gibson et al. [19]	0.950	Abd. CT	No
Linguraru et al. [10]	0.952 ± 0.014	Abd. CT	Yes

## Data Availability

The Medical Segmentation Decathlon (MSD) used as the “open dataset” in our study can be downloaded from http://medicaldecathlon.com (downloaded on the 12 March 2021). The in-house dataset cannot be made publicly available for data protection reasons.
